# Bridging the gap between in vitro and in vivo models: a way forward to clinical translation of mitochondrial transplantation in acute disease states

**DOI:** 10.1186/s13287-024-03771-8

**Published:** 2024-05-31

**Authors:** David F. Bodenstein, Gabriel Siebiger, Yimu Zhao, Aaron J. Clasky, Avinash N. Mukkala, Erika L. Beroncal, Lauren Banh, Lili Aslostovar, Sonya Brijbassi, Sarah E. Hogan, James D. McCully, Mohadeseh Mehrabian, Thomas H. Petersen, Lisa A. Robinson, Melanie Walker, Constantine Zachos, MITO2i-MbD Mitochondrial Transplant Consortium, Sowmya Viswanathan, Frank X. Gu, Ori D. Rotstein, Marcelo Cypel, Milica Radisic, Ana C. Andreazza

**Affiliations:** 1 Department of Pharmacology and Toxicology, University of Toronto, Medical Science Building, Room 4211, 1 King’s College Circle, Toronto, ON M5S 1A8 Canada; 2 Institute of Medical Science (IMS), University of Toronto, Toronto, Canada; 3 Latner Thoracic Research Laboratories, Toronto General Hospital, Toronto, Canada; 4 Institute of Biomedical Engineering, University of Toronto, Toronto, ON M5S 3G9 Canada; 5 Department of Chemical Engineering and Applied Chemistry, University of Toronto, Toronto, Canada; 6 Keenan Research Centre for Biomedical Science, Unity Health Toronto, Toronto, Canada; 7 Osteoarthritis Research Program, Division of Orthopedic Surgery, Schroeder Arthritis Institute, University Health Network, Toronto, Canada; 8 Krembil Research Institute, University Health Network, Toronto, Canada; 9 Centre for Commercialization of Regenerative Medicine, Toronto, Canada; 10 Mitochondrial Innovation Initiative (MITO2i), Toronto, Canada; 11 Regenerative Medicine Department, United Therapeutics Corporation, Silver Spring, USA; 12 Harvard Medical School, Boston, USA; 13 Department of Cardiac Surgery, Boston Children’s Hospital, Boston, USA; 14 Program in Cell Biology, The Hospital for Sick Children Research Institute, Toronto, Canada; 15 Department of Neurological Surgery, University of Washington, Seattle, USA; 16 Acceleration Consortium, University of Toronto, Toronto, ON Canada; 17 Li Ka Shing Knowledge Institute, Unity Health Toronto, Toronto, Canada; 18 Department of Surgery, University of Toronto, Toronto, Canada; 19 Toronto Lung Transplant Program, Division of Thoracic Surgery, Department of Surgery, University Health Network, University of Toronto, Toronto, ON M5G 2C4 Canada; 20 Toronto General Hospital Research Institute, University Health Network, Toronto, ON M5G 2C4 Canada; 21 Terence Donnelly Centre for Cellular and Biomolecular Research, University of Toronto, Toronto, ON M5S 3E1 Canada; 22 Department of Psychiatry, University of Toronto, Toronto, ON Canada

**Keywords:** Mitochondria, Mitochondrial transplant, Organ-on-a-chip, Joint-on-a-chip, Ischemia–reperfusion injury

## Abstract

Mitochondrial transplantation and transfer are being explored as therapeutic options in acute and chronic diseases to restore cellular function in injured tissues. To limit potential immune responses and rejection of donor mitochondria, current clinical applications have focused on delivery of autologous mitochondria. We recently convened a Mitochondrial Transplant Convergent Working Group (CWG), to explore three key issues that limit clinical translation: (1) storage of mitochondria, (2) biomaterials to enhance mitochondrial uptake, and (3) dynamic models to mimic the complex recipient tissue environment. In this review, we present a summary of CWG conclusions related to these three issues and provide an overview of pre-clinical studies aimed at building a more robust toolkit for translational trials.

## Background

Mitochondria are compartments within cells that regulate and mediate key biochemical pathways essential for many aspects of cellular function and biology. Additionally, mitochondrial dysfunction is known to contribute to a variety of diseases, ranging from primary mitochondrial disorders and chronic pathologies such as diabetes, heart failure, and Alzheimer’s disease, to acute conditions associated with ischemia–reperfusion injury (IRI), such as acute coronary syndrome, stroke, and pulmonary embolism [1]. All of these can benefit from cellular therapies as new and novel ways of treating disease. The mitochondrial innovation initiative (MITO2i) and Medicine by Design (MbD), two strategic initiatives of the University of Toronto, have brought together a world-renowned group of scientists and clinicians to create the Mitochondrial Transplant Convergent Working Group (CWG). This group came together with members of the University of Toronto and affiliated research hospitals, as well as external collaborators and partners, to discuss feasibility and techniques that can advance mitochondrial transplantation for successful applications in acute and chronic disease and regenerative medicine. In this first manuscript in a series of papers, we will focus on an overview of mitochondrial transplantation, explore biomaterials to enhance storage and delivery of mitochondria for mitochondrial transplantation for acute diseases, and propose approaches to bridge the gap between in vitro and in vivo models to help advance the clinical translation of mitochondrial transplant.

To accomplish these goals, we propose to further existing collaborations and to develop plan-to-action to accelerate the implementation of clinical mitochondrial transplant as a therapy of tissue regeneration for disease treatment. Mitochondrial transplantation is defined as the addition of live and healthy mitochondria retrieved from unaffected tissue or cellular models, like induced pluripotent stem cells (iPSCs), into target organs or tissues subjected to or prone to injury. Members of the CWG have expertise in mitochondrial function and stability, iPSC development, biomaterials for cell delivery, and clinical applications in regenerative medicine. The team includes researchers, clinicians, foundations, patients, patients’ families and caregivers, and industry partners—which together have a common objective of advancing mitochondrial related therapies to treat and cure disease.

## Overview of mitochondrial isolation, transplant, and biomaterials

Mitochondria are ubiquitous across cell types and serve an important role in the regulation and maintenance of homeostasis. Mitochondria differ from other organelles as they contain their own mitochondrial DNA (mtDNA) that encodes 37 genes, specifically 22 tRNAs, 2 rRNAs, and 13 protein subunits of the electron transport chain (ETC) [2]. The remaining mitochondrial proteins are encoded in the nuclear DNA (nDNA) and imported into the mitochondria through recognition of a mitochondrial targeting sequence [3]. This unique crosstalk of mtDNA and nDNA is further complicated by different mitochondrial haplogroups, which play a crucial role to ensure compatibility between the two genomes [4]. This is especially important in the development of heterologous mitochondrial transplantation and transfer.

Mitochondrial transplant and transfer are being explored clinically as a novel therapeutic approach in both acute and chronic diseases. Autologous mitochondrial transplant is being evaluated in acute diseases. It has been widely studied in animal models of ischemia–reperfusion injury, but also in children with congenital heart failure [5] and adult patients with cerebral ischemia (NCT04998357, currently recruiting patients) [6], as reviewed in D’Amato et al. [1]. In both studies, healthy mitochondria are isolated from non-ischemic skeletal muscle at the surgical access site and delivered either via direct injection or through intravascular infusion to the target site. Emani et al. [5] delivered 10 injections of 1 × 10^7^ mitochondria into the affected myocardium of pediatric patients with acute cardiogenic shock requiring extracorporeal membrane oxygenation (ECMO) support following cardiac surgery. Following mitochondrial transplant, four of five patients were successfully removed from ECMO with the fifth patient unable to be decannulated [5]. Of the four patients removed from ECMO support, one patient died later at 4 months old due to respiratory failure [5]. However, the exact therapeutic mechanism is not currently known and is a current limitation in the clinical advancement of mitochondrial transplant. Studies by Kesner et al. [7] and Pacak et al. [8] indicate that mitochondria are engulfed by endocytosis or macropinocytosis depending on the recipient cell type. Additionally, once the mitochondria are engulfed it is unknown how the mitochondria escape the endosomes to integrate into the host mitochondrial network. Cloer et al. [9] proposed that mitochondrial transplant upregulates autophagy, which reduces pro-inflammatory signalling and reactive oxygen species as well as providing additional metabolites for energy production. Notably, this is similar to the endogenous response of stressed or injured cells to release damaged mitochondria for degradation by surrounding cells, known as transmitophagy [10], to initiate mitochondrial biogenesis, immune and tissue repair responses by macrophages, and transfer of healthy mitochondria to restore cellular homeostasis [11, 12].

Mitochondrial transfer-based therapies are also being developed by Minovia Therapeutics Ltd. and IMEL Biotherapeutics Inc. for treatment of chronic diseases, such as primary mitochondrial disease [13, 14]. These methods differ from mitochondrial transplant as they rely on the ex vivo uptake of isolated mitochondria followed by intercellular transfer of healthy donor mitochondria into the target tissue. Briefly, hematopoietic stem and progenitor cells (HSPC) are isolated from patients and enriched with isolated healthy donor mitochondria [13, 14]. The enriched HSPC are then reinfused into the patient, after which donor mitochondria are transferred from the enriched HSPC to the target tissue through tunnelling nanotubules and extracellular vesicles to restore mitochondrial function [13, 14]. However, in acute diseases, such as those associated with IRI, where treatment must be delivered rapidly, this technique may not be applicable due to the time to isolate, enrich, and reinfuse autologous HSPCs, as well as to allow initiation of intercellular transfer of mitochondria to the target tissue. The direct transplantation of autologous mitochondria, however, ensures treatment can be delivered both effectively and rapidly to the target organ. Although it is important to note that there are several endogenous pathways to restore mitochondrial function and health following injury by intercellular transfer of mitochondria from mesenchymal stromal cells (MSC) [15, 16]. Mitochondrial transfer has also been suggested as a central mediator of the therapeutic efficacy of MSC-based therapies, as reviewed in Mukkala et al. [16].

Whether using mitochondrial transfer or transplant techniques, it is essential to first isolate pure and functional mitochondria from either tissue or cellular sources. There are several methods to isolate mitochondria, specifically ultracentrifugation, differential centrifugation, differential filtration, and kit-based methods. Ultracentrifugation and differential centrifugation both rely on multiple centrifugation steps to remove cellular debris and purify mitochondria usually through low- and high-speed spins [17]. Ultracentrifugation-based isolation methods can also be paired with a density-gradient to further separate non-synaptic and synaptic mitochondria from brain homogenates [17]. However, functional assays of the isolated mitochondria are hindered due to the long isolation times of both methods. Preble et al. [18] developed a differential filtration method which removes the need for multiple centrifugation steps, reducing the isolation time to approximately 30 min. Briefly, cell homogenate is filtered through 40, 10, and 5 μm filters to gradually remove cellular debris and organelles, followed by a final high-speed centrifugation to concentrate the isolated mitochondria [18]. Magnetic bead-based isolation methods have been commercialized by Miltenyi Biotec; however, these are only applicable in the laboratory setting (Catalogue No: 130-094-532, Miltenyi Biotec). Current clinical studies in mitochondrial transplant for pediatric post-cardiotomy cardiogenic shock and cerebral ischemia use differential filtration to isolate pure and functional mitochondria prior to transplantation [5, 6].

### Biomaterials to enhance donor mitochondrial uptake and integration

Biomaterials such as polymers and lipids have recently been investigated to facilitate the delivery of isolated mitochondria to tissues of interest (Table 1). These materials are commonly used to improve the bioavailability of various payloads, including small molecule drugs and proteins, in different configurations such as nanoparticles [19] and hydrogels [20], and have been tested rigorously for biocompatibility and efficacy in animal models. Numerous formulations are currently moving towards human clinical trials [21] and many of the materials mentioned in this section have FDA approval for at least one application, including: Pluronic F-127 [22], hyaluronic acid [23], poly(ethylene glycol) [24], chitosan [25] and methylcellulose [26].

**Table 1:** Potential biomaterials to enhance cellular uptake of isolated mitochondria

Biomaterial	Abbreviation	Mitochondrial coating	Purpose
Hyaluronic acid	HA	Patel et al. [33]	Mimicking extracellular matrix
Methylcellulose	MC	Patel et al. [33]	Thermogelling
Pluronic F127	PF127	Huang et al. [32]	Protect and enhance mitochondrial uptake
Poly(N-isopropylacrylamide)	PNIPAAm		Temperature response
Poly(pyrrole)			Electrical conductivity
Alginate			Ion response
Poly(ethylene glycol)	PEG	Chen et al. [36]	Hydrophilicity
Poly(vinylpyrrolidone)	PVP		Hydrophilicity
Cyclodextrin-based polymer	β-CD		Injectable and self-healing properties
Poly(vinyl alcohol)	PVA		Hydrophilicity
Poly(acrylic acid)	PAA	Chen et al. [34]	Water retention, pH sensitivity
Poly(2-hydroxyethyl methacrylate)	PHEMA		Inhibit cell adhesion
Dioleoyl-3-trimethylammonium propane	DOTAP	Nakano et al. [35]	Artificial membrane
1,2-dioleoyl-*sn*-glycero-3-phosphoethanolamine	DOPE	Nakano et al. [35]	Artificial membrane
Triphenylphosphonium-conjugated dextran	Dextran-TPP	Wu et al. [28], Liu et al. [29], Alexander et al. [30]	Enhance mitochondrial uptake

In the context of mitochondrial transplantation, biomaterials have been shown to improve the stability of mitochondria in solution and to improve cellular internalization to ameliorate therapeutic outcomes. Hydrophilic cationic polymers and/or lipophilic moieties are normally used to maximize interactions with both the negatively charged mitochondrial surface and membranes of the target cells [27]. Triphenylphosphonium-conjugated Dextran (Dextran-TPP) has emerged as a common coating material for mitochondria [28–30]. Dextran is a polysaccharide that has been shown to improve uptake of nanomaterials in vivo and TPP is a cationic, lipophilic ligand that readily associates with mitochondrial membranes and can be easily conjugated to the Dextran backbone [28, 31]. Dextran-TPP improves the cellular uptake of coated mitochondria post-transplant, likely due in part to the change in surface properties (-44 mV surface charge for bare mitochondria compared to just -4 mV for Dextran-TPP-coated mitochondria) [28]. This improved cellular uptake and transplantation efficiency also enables similar outcomes to be achieved with much lower dosages: for example, nasally administered Dextran-TPP mitochondria reversed chemotherapy (cisplatin)-induced cognitive defects and resolved neuropathic pain at a dose 55-times lower compared to bare mitochondria [30]. Literature also suggests that the Dextran-TPP coating induces a state of metabolic dormancy in the mitochondria (based on a reduced respiratory control ratio and reduced LEAK state), likely by restricting entry of substrate into the mitochondria [28]. Since metabolic activity is fully recovered following transplantation of the coated mitochondria, the dormant effect may potentially be used to preserve the isolated mitochondria for longer periods of time. It is currently unclear whether this metabolic dormancy has been observed in other coating systems and to what extent it influences the preservation and long-term viability of isolated mitochondria. Future work on polymer-mitochondria systems may look to consider this effect in more detail.

Different polymers have also been investigated for mitochondrial delivery. Pluronic F127, an amphiphilic block copolymer of poly(ethylene oxide)_98_-poly(propylene oxide)_67_-poly(ethylene oxide)_98_ (PEO_98_-PPO_67_-PEO_98_), was used to coat mitochondria for treatment of myocardial IRI [32]. The coating was shown to improve membrane integrity in a high-Ca^2+^ simulated transplant environment and improve cellular uptake. Similarly, a combination of hyaluronic acid (HA) and methylcellulose (MC) was used to produce a thermogelling hydrogel to deliver mitochondria for spinal cord injury treatment [33]. It should be noted, however, that this latter system is composed of a hydrogel containing mitochondria rather than a coating for individual mitochondria, although there is still an improvement in viability (oxygen consumption rate (OCR) over time) and uptake, with the added potential benefit of prolonged mitochondrial release compared to injection. More complex approaches include a layer-by-layer strategy which leveraged the opposing charges of chitosan (positive) and poly(acrylic acid) and the mitochondrial membrane (negative) to produce a tunable multilayered coating to combat multidrug resistance in cancer treatment [34]. In addition to overcoming issues with electrostatic repulsion, the layer-by-layer method also embedded siRNA for P-glycoprotein to overcome drug resistance through knockdown of drug transporters [34]. The intracellular ion-rich environments trigger dissociation of the electrostatic layers, thus releasing siRNA and intact mitochondria at the site of action for synergistic treatment. In addition to polymer systems, lipid bilayers consisting of cationic dioleoyl-3-trimethylammonium propane (DOTAP) and 1,2-dioleoyl-*sn*-glycero-3-phosphoethanolamine (DOPE) were formed around isolated mitochondria using an inverse emulsion method to produce “artificial membranes” [35]. These lipid bilayer coatings improved neuroprotection in cultured neurons and amplified cerebroprotection in-vivo after focal cerebral IRI in mice compared to uncoated mitochondria [35].

In addition to supporting mitochondrial transplantation, biomaterial coatings can also confer additional capabilities to the mitochondria. Janus-like coatings were produced via immobilization of mitochondria on a glass slide with one side containing a deposition of alternating layers of chitosan and PAA with a covalently grafted poly(ethylene glycol) (PEG) brush, and the other side containing glucose oxidase (GOx) [36]. The one-sided GOx enables the mitochondria to follow the glucose gradient within a tumour via chemotaxis and penetrate much deeper compared to either uncoated or fully GOx-coated mitochondria. This strategy resulted in significantly more accumulation in cultured mammospheres and longer retention time in vivo compared to controls. Mitochondria can also be functionalized with lipid-modified photosensitizers to combine mitochondrial transplantation with photodynamic therapy for cancer treatment [37, 38]. The potential of biomaterials to accelerate the utility of mitochondrial transplantation is significant: from synergistic combinations with excipient drugs to stimulus-responsive release and activation strategies.

A limitation with current mitochondrial transplant methods is the short lifespan of isolated mitochondria, which significantly lose respiratory function after approximately 2 h [9, 39]. It is essential to use viable and functional mitochondria during transplantation, as non-viable or damaged mitochondria may release damage associated molecular patterns (DAMPs) leading to immune activation [40]. Furthermore, non-viable mitochondria were found to have no cardiac protection in rabbits following 30 min of regional ischemia [41]. Therefore, with current approaches, this means that mitochondrial isolations must ideally be performed at the bedside prior to transplantation to minimize decreases in function/viability of isolated mitochondria, thereby optimizing function. Yamaguchi et al. [42] developed a method to store isolated mitochondria in a modified buffer containing trehalose as a cryoprotectant. Mitochondria stored in this solution retained mitochondrial outer membrane integrity, response to Bcl-2 family proteins, calcium-induced swelling, ATP synthesis, transmembrane potential (MP), and transmembrane mitochondrial transport similarly to freshly-isolated mitochondria in isolated mitochondria from mouse liver [42]. Bioenergetic function, when analyzed in terms of both phosphorylating state [3] and maximally uncoupled respiration, however, was shown to be decreased as compared to freshly isolated mitochondria, whereas activity of cytochrome C oxidase was intact. The authors’ conclusion was that the solution was capable of partially preserving mitochondrial function, to the extent of retaining MP and MP-dependent functions, including ATP synthesis, protein import and calcium accumulation leading to opening of the mitochondrial permeability transition pore (mPTP).

By the analysis of Cloer et al. [9], trehalose-frozen mitochondria obtained by a manual homogenization, centrifugation-based isolation method retained equivalent ultrastructural morphology, size, complexity, membrane potential, permeability, and basal and maximal respiration to freshly isolated mitochondria. Acute antioxidant functionality was maintained in frozen compared to freshly isolated mitochondria but an in vivo comparison between the storage conditions was not reported. Following a similar protocol, Cloer et al. [9] observed no changes in functional or morphological assessments in isolated mitochondria stored at – 80 °C for up to 12 months. The ability to isolate and store mitochondria at – 80 °C would allow users to perform complete quality control assessments on isolated mitochondria prior to transplantation in injury models and predictable ischemic events. This would ensure mitochondrial transplants are performed with high purity and functionally viable mitochondria to reduce the risk of adverse effects and improve patient safety. Frozen mitochondria retained functional benefits post-mitochondrial transplant in porcine lungs [9].

### Functional and safety checkpoints of mitochondrial isolation and transplant

After isolating and transplanting mitochondria, purity and function in the recipient tissue/cell can be evaluated using mtDNA content, western blot, ATP production, and OCR. mtDNA content is measured by qPCR using mtDNA and nDNA specific primers, allowing researchers to determine the number of copies of mtDNA per nDNA [43, 44]. In whole cells or tissue, this provides insight into mitochondrial biogenesis, which is an important part of the mitochondrial stress response pathway, along with mitochondrial fission, fusion, and mitophagy to maintain mtDNA integrity [45, 46]. Following ischemic injury, these pathways will be activated to remove damaged mitochondria and induce mitochondrial biogenesis to restore and maintain mitochondrial function [46]. Alternatively, in isolated mitochondria, mtDNA content allows researchers to assess mitochondrial isolation purity as minimal nDNA should be amplified. Mitochondrial purity can also be assessed using western blot and flow cytometry for common organelle contaminants, such as endoplasmic reticulum, peroxisome, and lysosome [47, 48]. It is essential to ensure maximal mitochondrial isolation purity as any nDNA or other organelle contamination can lead to adverse immune activation during mitochondrial transplantation [47]. Kit-based ATP production assays and OCR are useful tools for evaluating mitochondrial function post-isolation and treatment efficacy post-mitochondrial transplant [47].

It is necessary to assess the safety profile of the mitochondrial transplant by measuring immune response, cellular viability, and mitochondrial function. Damaged mitochondria release mtDNA and cytochrome c, which are recognized as DAMPs, activate the NLRP3-inflammasome and apoptosis [40]. Common assays such as MTT, picogreen, DCFH-DA, and Griess assay are easily performed to assess cellular viability, double strand DNA (dsDNA) release, and reactive oxygen/nitrogen species (ROS, RNS) formation, respectively [49]. Furthermore, IL-1β and caspase-1 can also be assessed at the same time to evaluate the activation of the NLRP3-inflammasome signaling cascade [40]. This workflow is ideal as the mitochondrial transplant can be performed in a well-plate format for MTT assay and the cell media can be collected to perform all other assays, allowing the results to be normalized to cellular viability [49].

The ideal mitochondria dosage and metabolic profile must also be carefully considered in ongoing in vitro and in vivo studies. Zhang et al. [50] demonstrated that cellular engulfment of exogenous mitochondria is unchanged with increasing mitochondrial concentrations. Similarly, Shin et al. [51] found that delivery of mitochondria above 2 × 10^6^ mitochondria/gram of wet tissue resulted in no greater therapeutic efficacy or cardioprotective effect in porcine models of myocardial ischemia–reperfusion injury. Furthermore, Zhang et al*.* [50] also identified the importance of metabolically matching donor mitochondria to the recipient tissue to ensure maximal therapeutic efficacy. Specifically, mitochondrial transplant of metabolically matched mitochondria into neonatal mouse cardiomyocytes was necessary to restore maximal OCR and contractility following doxorubicin-induced myocardial dysfunction. Therefore, in future studies it is important to consider both the mitochondrial concentration to ensure the recipient tissue is not overwhelmed with donor mitochondria leading to adverse effects and the metabolic matching of donor and recipient mitochondria for maximal therapeutic efficacy.

Depending on the method of administration, the coating and delivery of transplanted mitochondria may be complicated by the presence of endogenous proteins and other components in the body. Many of the materials used to coat mitochondria are common in nanomedicine formulations and their interactions with proteins in complex biological media are the subject of significant interest [52–54]. The layers of proteins formed on the surface of a nanomaterial are referred to as the “protein corona” and may be influenced by both the biological medium and the outer coating material [55, 56]. In addition to changing the outer surface of the (coated) mitochondria, protein adsorption may interfere with cellular uptake and potentially trigger immune responses [57]. Further development of biomaterial-coated mitochondria also requires consideration of their interactions with other components in complex biological media and how these interactions may influence any therapeutic outcomes.

Compatibilities between the donor and recipient are important considerations to avoid immune complications or reactivities. mtDNA-nDNA crosstalk and energy metabolism compatibilities through mitochondrial haplogroups are important to be considered, especially in mitochondrial transplantation, as several studies reveal the importance of mitochondrial function in reducing post-graft dysfunction and improving transplant success [58, 59]. mtDNA haplogroup influences energy metabolism, production of ROS, and OCR [60, 61]. These differences in haplogroups suggest the importance of genetic compatibility to improve the adaptation of the transplanted mitochondria to their new environment. A study on conplastic mice demonstrates that mismatch of mtDNA variants is enough to promote differences in mitochondrial function and cellular adaptive responses [4]. This is due to the adaptive response led by a complex network of mitochondrial stress pathways that impact mitochondrial proteostasis, mtUPR, and ROS signaling, affecting the organism’s metabolic performance [4]. For example, haplogroup H has an increased risk for chronic renal allograft dysfunction while haplogroups V and J have lower risks. This was explained by the increased activity in the ETC in haplogroup H, resulting in increased ROS production [62]. Therefore, current data emphasizes the importance of considering haplogroups when performing heterologous mitochondrial transplantation.

## Uses of mitochondrial transplant in acute illness

### Ischemia–reperfusion injury

Ischemia–reperfusion injury (IRI) is defined as the series of pathophysiological alterations that paradoxically occur in tissues upon reperfusion following the deprivation of blood supply, and is characterized by a rapid burst of ROS, which surpasses the tissue’s antioxidant capacity [63]. This leads to marked increase in cytokine, chemokine, and cell adhesion molecules’ release, and finally to the late recruitment of neutrophils, which further accentuates tissue damage [64]. Hayashida et al. [65], in a systematic review published in 2021, examined the evidence from human and animal studies in support of the safety and efficacy of mitochondrial transplantation for the treatment of IRI in different organ systems. Importantly, studies in ex-vivo models, however, were excluded from the analysis. Since then, important contributions to the field of mitochondriology have been made in ex-vivo perfusion platforms, particularly in models of heart and lung transplantation. In effect, the evaluation of mitochondrial transplant ex-vivo provides unique possibilities for both research and future clinical translation, both for safety and efficacy studies. By isolating the effect of mitochondrial transplant to a single organ (with no systemic escape), ex-vivo systems reduce or eliminate exposure to scavenging systems (such as the reticuloendothelial). Additionally, they allow the study of the interaction of mitochondria with different perfusion solutions in varying temperatures.

Although acute events of ischemia and their subsequent reperfusion injury can all be referred to as IRI, several distinguishing features should be taken into account when establishing mitochondrial transplant strategies: (1) the length of the ischemic insult (short vs extended ischemic times), (2) the type of ischemic insult (cold ischemia vs warm ischemia), and (3) the predictability of the ischemic insult (with heart transplantation as an example of a somewhat predictable insult, vs acute coronary occlusions as an example of unpredictable event). Predictable ischemic events provide the window for pre-ischemic organ conditioning, as well as allow time for autologous mitochondrial isolation for transplantation with less significant time constraints. These are important concerns because they affect the time points in which mitochondrial transplant could be potentially applied, the mechanism of injury of the affected organ, and the mechanism of protection that could potentially be obtained by mitochondrial transplant.

Different pathophysiological processes are encompassed by the broad terminology of “IRI” in other organs and are similarly dependent on the duration and type of ischemic insult. Fischer et al. demonstrated that cell death in lung tissue is dependent on the duration of cold ischemic time (CIT). Apoptosis occurs more frequent than necrosis in short periods of CIT (< 12 h), while necrosis occurs more in extended CITs (of up to 24 h) [66]. Furthermore, Iskender et al. [67] provided evidence in a rat model that lungs exposed to an extended CIT of 18 h compared to a warm ischemia time (WIT) of 3 h are injured very differently, despite presenting similar functional outcomes in terms of lung physiology and oxygenation. Warm ischemia resulted in higher plasmatic M65 levels (a marker of apoptotic and necrotic cell death), as well as higher levels of pro-inflammatory cytokines and chemokines in plasma, than both standard (12 h) and extended (18 h) CIT groups. Interestingly, similar injury markers were higher in the extended CIT group than in the WIT group when analyzed in tissue samples, suggesting divergent local and systemic responses. Finally, tissue ATP levels were the lowest in WIT lungs at the end of the ischemic period, but all groups (WIT, standard CIT, and extended CIT) demonstrated a *reduction* in ATP levels upon 2 h of reperfusion as compared to post-ischemic (and pre-reperfusion) measures. This indicates that even with the recovery of blood supply, local energetic demands to cope with the burst of ROS are higher than the parenchymal capacity. Therefore, the central importance of mitochondrial metabolism in lung IRI cannot be overemphasized.

### Organ transplant

Models of organ transplantation provide a relevant example of the importance of these concepts. In organ transplantation, CIT is defined as the period through which harvested tissues are subjected to deprivation of blood supply while maintained passively or actively at low temperatures. By keeping tissues cooled down, the expectation is the reduction of metabolic demand and the subsequent extension of organ viability [68]. For heart transplantation, CIT of 4 h or longer is associated with lower recipient survival, primarily due to IRI [69]. Furthermore, transplanted organs are always subjected to periods of *warm* ischemia, out of cold preservation solutions, referred to as WIT [70]. This includes ischemia during organ retrieval and during implantation in the recipient. More recently, the advent of organ donation after circulatory death (DCD), aimed at increasing the donor pool, generated a third period of WIT impacting the quality of some donated organs, including hearts [71]. In the heart, it is known that WITs longer than 10 min increase mitochondrial damage and compromise ETC activity, increase caspase 3 and 7, induce cardiomyocyte apoptosis, and decrease overall myocardial function and primary graft failure [72]. Due to the nature of DCD, which by legislation requires a no touch period of variable duration (typically 5–20 min), pre-emptive pre-ischemic conditioning through mitochondrial transplant would not be feasible [73].

## Regenerative medicine models to aid in studying mitochondrial transplant

### In vitro models

In vitro models are systems established outside of the human body to study human biological functions. Compared to animal models that can provide systemic simulation, in vitro models aim to distill the complexity of human (patho)physiology into key biological events and answer questions of interest. As in vitro culture eliminates species-related physiological differences by using human primary and stem cell-derived cell sources, they can significantly complement the results from animal models for initial exploratory or follow-up validation studies. More importantly, these in vitro models are typically cost-effective, have high throughput, and have low ethical liability, which facilitates the discovery process for disease mechanisms and drug leads.

**Fig. 1:**
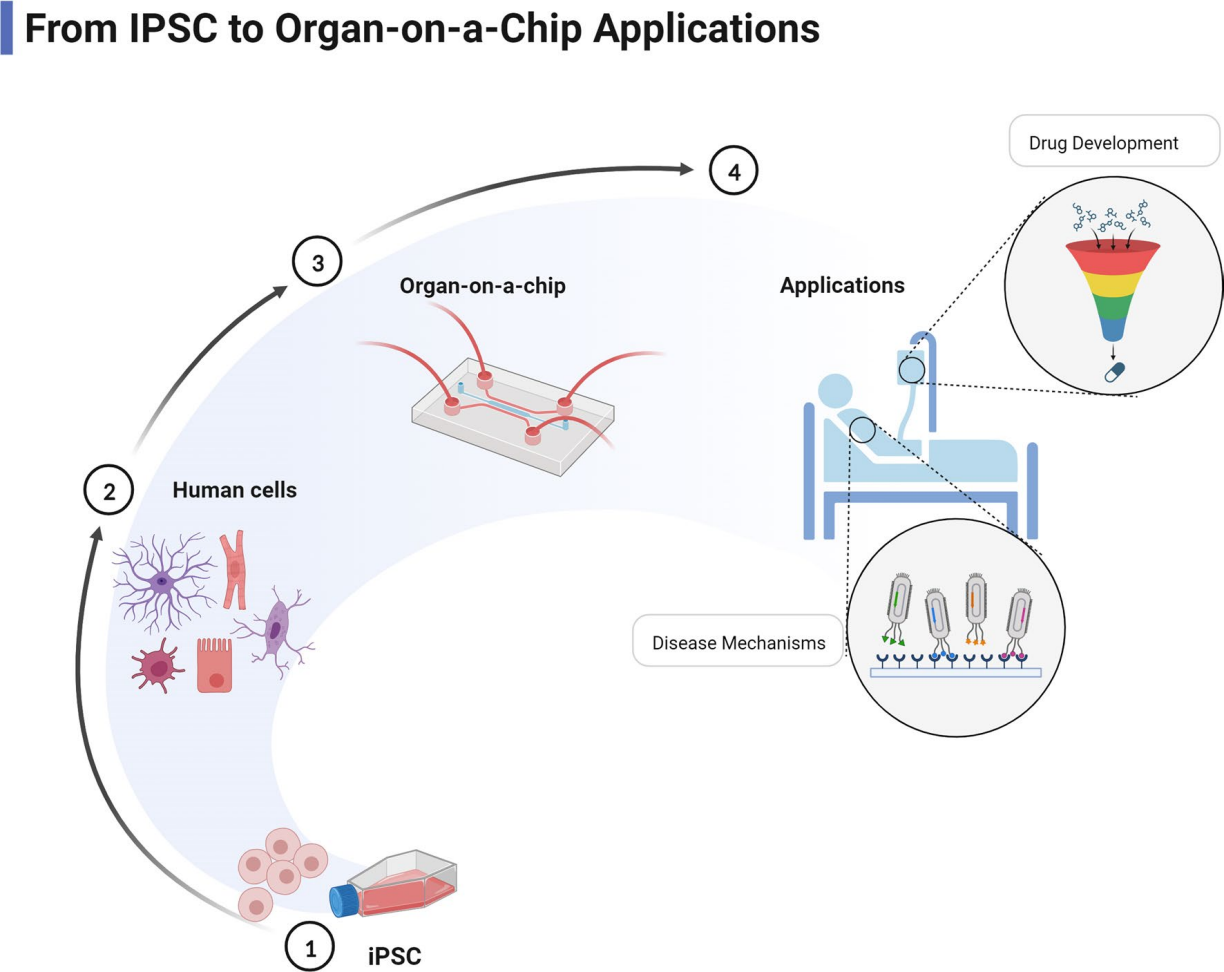
Overview of the generation of organ-on-a-chip models from iPSCs and their downstream applications. iPSCs are reprogrammed from patient PBMCs or fibroblasts by transfection with OCT4, SOX2, KLF4, L-myc, and Lin28. Following iPSC stabilization, cells are differentiated to desired cell type and seeded on biomaterials and scaffolds to generate organ-on-a-chip models. Organ-on-a-chip models mimic in vivo tissue by replicating intercellular signalling and microenvironment to generate advanced in vitro disease models. Figure created with BioRender.com

The current in vitro models for mitochondrial study are limited to the use of single cells or monolayer cells, as they permit the observation of mitochondria entrance and colocalization [7]. However, there are many well-established in vitro models, ranging from simpler single-cell cultures to more complex tissue-engineered organ models. Patient-derived iPSCs present a unique opportunity to develop 3D organoids and organ/joint-on-a-chip (OoC; JoC) models to evaluate drug and treatment efficacy (Fig. 1). These models allow the recapitulation of key tissue phenotypes and have been developed for single organ and multi-organ models [74, 75]. Peripheral blood mononuclear cells or fibroblasts can be collected from patient blood or skin biopsy to reprogram into iPSCs via transfection with pluripotency factors, specifically Sox2, Oct4, Klf4, L-myc, and Lin28 [74]. Once stabilized, these iPSCs can be differentiated into 2D cellular models, e.g. cortical neurons [76], myofibers [77], and cardiomyocytes [77], or 3D cellular models, e.g. cerebral organoids [74, 78], cardiac tissue [79], and other OoC/JoC models.

The experimental throughput, associated costs, and user accessibility suffer with the increased complexity of tissue models. Yet, more complex models better recapitulate the in vivo microenvironment and provide clinically relevant results. For example, 3D tissue models are considered superior to 2D cultures in terms of replicating in vivo-like cell–cell and cell–matrix interactions, as well as cellular morphology, arrangement, and genetic and protein expressions [80]. However, simple 3D tissue models, such as cell aggregates, often oversimplify the physiological microenvironment observed in vivo, limiting their translational potential. Furthermore, mitochondrial maturity, morphology, and function change with the degree of cell differentiation as iPSCs shift to oxidative phosphorylation during differentiation [74, 81]. Therefore, it is necessary to assess multiple cell types in models that have mature mitochondria. The use of OoC and JoC technology allows researchers to examine multiple tissues and cell types with mature mitochondria. Previously, this work had to be performed in animal models, which are time-consuming and expensive to work with and may not translate to humans. Ethical considerations must also be considered for animal studies. To overcome these challenges, organoids, OoC, and JoC technologies were developed to better address clinically relevant questions.

Organoids, OoC, and JoC technologies are gaining increased attention in biomedical research due to their advantages of building clinically relevant, high-throughput, and high-fidelity in vitro models. Organoids, often derived from iPSCs or adult organ progenitor cells through embryogenesis, can self-differentiate into three-dimensional structures that mimic the cellular diversity and architecture of human organs [80]. This ability to recapitulate complex human tissues lends a unique level of clinical relevance using organ-specific organoids [80]. For example, lung organoids derived from human iPSCs obtained structural polarity with minimally 7 key cell types out of the 40+ cell types in the lung[82]. On the other hand, OoC devices employ microfabrication technology to culture cells in a controlled 3D microenvironment, utilizing human stem cell derived sources, biological scaffolding, and chemical and biophysical cues, to mimic human organ-level functions, making them indispensable for (patho)physiological studies [83]. Electrical, chemical, and biological cues can be added to promote the functional maturation towards the adult phenotypes which further increases the clinical fidelity [84, 85]. More importantly, OoC devices typically integrate built-in functional sensors, which provide non-invasive functional readouts corresponding to organ-specific functions or various experimental designs [77]. Heart-on-a-chip, as an example, often uses elastomer-based materials as tissue guiding templates and force sensors for the continuous force of contraction readouts. The electrical activity of the neural tissues can also be recorded using MEA-integrated devices [86].

While organoids and OoC devices offer numerous advantages, it is crucial to acknowledge that these tissue models aim to recapitulate only minimal tissue or organ function, often without considering relative scales [87]. The input cell populations are frequently reduced to several major cell types to mitigate complex experimental designs and exponentially increasing costs. Additionally, the media-to-cell ratio is often over-scaled, resulting in overly diluted chemical cues compared to native tissue. These inconsistencies partially stem from insufficient tissue vascularization and artificial methods of nutrient/oxygen delivery. To date, it is still challenging to establish a long-lasting, functional vascular network embedded with parenchymal tissues that faithfully recapitulates the vascular density and dynamic nature. Moreover, these organoids and OoC tissue models must undergo extensive validation at both functional and molecular levels to demonstrate tissue fidelity, which is the foundation of using these platforms to predict the outcomes of clinical study. These limitations could potentially impact downstream experimental designs concerning therapeutic agents’ delivery methods, concentrations and efficiency, such as mitochondrial transplantation, from OoCs to human studies in the future. Without this requirement, the OoC simply serves as a proof-of-concept tool and requires further modifications to be applicable in future studies.

### Key design criteria of in vitro platform for mitochondrial transplantation

To tune these platforms specifically toward studying the mechanisms of mitochondrial transplantation, the following design criteria are pivotal for success. First, the tissue models should be constructed from human cells and require a high level of functional maturation and can recapitulate the key responses after injury. Using heart-on-a-chip as an example, iPSC-derived cardiomyocytes can be very immature and do not have high metabolic activity, highly organized intra-cellular sarcomere ultrastructure, or efficient machinery for cardiac output. In the study carried out by Ronaldson-Bouchard et al. [84], after electrical conditioning, the cardiomyocytes have 30% volumetric occupancy of mitochondria, compared to 10% in immature tissues. As a result, these immature cardiomyocytes can be much more resilient to ischemia–reperfusion injury compared to the high-metabolic demanding, highly functional matured cardiomyocytes [88]. Thus, the therapeutic effect of mitochondrial transplantation cannot be fully evaluated in these models. Secondly, the presence of immune cells can be an important factor. Both tissue-resident macrophages and bone marrow-derived macrophages play important roles in tissue injury and remodeling [89]. The incorporation of these cell types can be critical to recapitulate the cellular interactions during the injury and healing process, as they could be the first responders to the transplanted mitochondria. As the sources of the mitochondria can be allogenic for in vitro experiments, potential innate immunological responses can be anticipated and can be modeled with the presence of relevant immune cell types. Although the local transplantation of mitochondria onto the injury sites is the most used method in vivo. The incorporation of blood vessels can be useful to apply the transplantation through intravascular infusion in tissue models. Thus, the incorporation of the vascular system can be useful to investigate how mitochondria travel through the vascular system, surpass the vascular barrier, then localize at the injury sites and penetrate the parenchymal tissue.

### Injury models using organoids and OoC technologies

Since most of recent research on mitochondrial transplantation has been focused on its potential for the treatment of IRI, it is important to understand how such a model could be achieved when making use of OoC technologies. The typical IRI model consists of applying a small volume of ischemic media with minimal nutrients and/or a low oxygen environment to the healthy tissues for a significant period, allowing metabolic waste accumulation to mimic nutrient deprivation, hyperkalemia, high lactate concentration, and low extracellular pH that are commonly observed during ischemia in the local tissues [88]. The following reperfusion step can be chosen to restore the environment to normal oxygen, nutrient, and pH levels by changing back to the normal culture conditions and supplemented media to model reperfusion injury [88, 90–92]. For cardiac IRI on-chip model, the cardiac microtissues were made from iPSC-derived cardiomyocytes and cardiac fibroblasts [88]. The OoC platform allowed in-situ functional readouts, i.e., force of contraction, by video tracking the biomaterial-based force sensors. The results suggested that reperfusion injury had more significant functional reduction compared to ischemic injury [88]. Additionally, functional more matured tissues experience more cellular damage compared to their immature counterparts [88]. In a separate heart-on-a-chip study, extracellular vesicles were shown to mediate the IRI damage on the heart, demonstrating the potential therapeutic effect of mitochondrial transplantation [92]. In a neurovascular IRI stroke models, brain endothelial cells showed reduced barrier function and decreased mitochondrial potential as well as reduced ATP in both the blood- and the brain side of the model, suggesting the effective injury of the tissue model upon hypoxia [91]. Similarly, kidney on-a-chip [90] was also used to demonstrate the IRI injury. In this study, the proximal tubule epithelial cells were in direct co-culture with blood vessels and demonstrated tight barriers with perfusion. The ischemia injury was induced through a non-flow, no-glucose condition. The epithelial depletion and cell death were observed during the reperfusion stage, whereas adenosine treatment was shown to mediate the renal ischemic injury.

OoC and JoC are far from replicating the entirety of native organ/tissue complexity. However, the reductionist approach in which hallmark features are exclusively replicated minimizes potential design complications and results in a valuable tool that can be used in multiple applications. These dynamic devices are an additional tool to evaluate the effects of novel therapeutics and raise the odds that therapies will successfully move from pre-clinical to clinical studies [75, 93, 94]. Patient specific iPSC-derived OoC and JoC can be used to understand disease pathology via tissue engineering approaches to model longitudinal disease progression. These models can also be used to screen drug candidates and other therapies to select effective treatments for further testing in other preclinical models and patients [95]. This technology allows researchers to develop models for each patient and evaluate treatment efficacy under controlled laboratory settings, facilitating personalized medicine. Additionally, this aids in the development of new treatments, such as mitochondrial transplantation, as researchers can rapidly screen new biomaterials to enhance mitochondrial uptake and delivery in tissue and disease models.

### In vivo preclinical models of mitochondrial transplant in acute organ injury

*Heart*: Work by Guariento et al. [96] explored mitochondrial transplant in a porcine model of DCD, in which hearts were subjected to 20 min of WIT, with subsequent evaluation in ex-vivo heart perfusion. Mitochondrial transplant administered at 15 min of reperfusion (single dose) or at both 15 min and at 2 h of reperfusion (serial dose) by intracoronary administration were then compared to vehicle controls during 4 h of ex vivo heart perfusion (EVHP). Importantly, for the serial dose group, whilst the first application of mitochondria was autologous, the second was not, with mitochondrial extraction obtained from porcine cardiac fibroblasts. The authors reported that no inflammatory response, as shown by hematoxylin & eosin staining, could be observed in the serial mitochondrial transplant group, in accordance with previous data from the group, in which no proinflammatory, damage-associated molecular patterns, or allorecognition signals were observed with allogeneic mitochondrial transplantation [97]. Interestingly, no additional benefit was observed in the 4-h assessment period by adding a second mitochondrial transplant after 2 h of heart reperfusion, both in terms of improved organ function (such as contractility and developed pressures) and myocardial infarction area size. In pathway analysis of hearts that received a mitochondrial transplant, mitochondrial ETC was included in the top 10 pathways with altered metabolic profile [96]. Importantly, the authors observed no difference in ATP content of porcine mitochondria isolated after the 15-min no-touch period after cardiac arrest, as compared to isolations in non-ischemic skeletal muscle.

More recently, Alemany et al. [98] also explored a similar model of autologous mitochondrial transplant for DCD in pediatric and neonate Yorkshire pigs. The pediatric population (10–15 kg) and the neonate population (3–4.5 kg) hearts had comparable sizes to those of 4- to 6-year-old children and 5- to 11-month-old infants, respectively. In contrast to previous work, mitochondrial transplant was applied only as a single bolus, albeit in a similar count (5 × 10^9^ particles), and the ex-vivo heart perfusion machine was primed with blood donated from an adult female donor (non-autologous system priming). Similarly, to what had been observed with larger animals, mitochondrial transplant improved cardiac function and reduced infarction size as compared to vehicle controls. Intriguingly, slightly better function was noticed in the neonatal group (vs pediatric) in some parameters, which—as pointed out by the authors—could be resultant of a higher concentration of mitochondria per gram of tissue for neonate hearts under a fixed mitochondrial transplant dose. Additionally, contrasting what had been observed with larger animals, mitochondrial isolations performed in ischemic donor tissue showed reduced viability as compared to those obtained from non-ischemic tissue, which highlights that even for the same species, age-differences are non-negligible for mitochondrial transplant purposes, and dose extrapolations from previous studies (generally coming from McCully’s group data, targeting 2 × 10^5^ to 2 × 10^6^ particles/gram of wet tissue[41]) may not always result in similar efficacy.

Guariento et al. [99] demonstrated in a porcine model that intracoronary administration of autologous mitochondria, whether in a single bolus injection (1 × 10^9^ particles) or serial bolus injections over 60 min (1 × 10^9^ particles, 10 times), when given 15 min *prior* to ligation of the left anterior descending artery (LAD) in Yorkshire pig hearts, could significantly attenuate IRI after 30 min of regional ischemia. By assessing reperfused hearts for 2 h, without any additional mitochondrial transplantation upon reperfusion, the authors showed improved organ function, with higher contractility, enhanced ejection fraction and fractional shortening, and significant reduction in infarct size, regardless of the mitochondrial transplant strategy (single or multiple injections). Interestingly, coronary blood flow and contractility were significantly improved compared to baseline heart function even prior to the ischemic event. Additionally, these striking results demonstrated that, even when subjected to the same ischemic event as the target organ, transplanted mitochondria could still positively impact organ function and prevent infarction. The mechanism that explains how mitochondrial transplant operates on pre-ischemic heart conditioning still needs to be further explored and elucidated.

Moskowitzova et al. [100] explored in a murine model intracoronary administration of autologous mitochondria (1 × 10^8^ particles, obtained from the gastrocnemius muscle) at two combined time-points, the first of which 10 min prior to harvest, and the second 5 min following reperfusion after heterotopic transplantation in the recipient. For this study, an extended period of 29 h of CIT was used as the main injury model, and transplanted animals were functionally evaluated for 24 h. Implanted hearts subjected to mitochondrial transplant demonstrated improved contractility, reduced necrosis, and reduced neutrophil infiltration in comparison to vehicle controls. Preliminary data reported by the authors suggested that the dual-administration regimen (*n* = 8) was superior to a single dose preceding CIT (*n* = 2) in providing improvement in the Stanford Cardiac Surgery Laboratory graft scoring system[101], hence the decision to include two mitochondrial transplant procedures. No other studies addressing mitochondrial transplantation at a *single* time point *preceding* extended cold ischemic times in heart transplantation have been identified in the literature.

Stemming from the encouraging pre-clinical results of mitochondrial transplant in cardiac IRI, Guariento et al. [102] conducted the first non-randomized case series of mitochondrial transplants in pediatric patients requiring ECMO for postcardiotomy cardiogenic shock (ClinicalTrials.gov Identifier: NCT02851758). In addition to requiring ECMO, patients were required to present proven documented ischemic event followed by successful revascularization and moderate to severe, persistent systolic ventricular dysfunction. Ultimately, 10 patients underwent mitochondrial transplant through direct transepicardial injection of autologous mitochondria obtained from the rectus abdominis muscle. Patients were retrospectively compared to 14 controls who underwent no additional interventions apart from the inclusion criteria. Direct intramyocardial mitochondrial transplant resulted in significantly improved successful separation from ECMO support (80 vs 29%, *p* = 0.02), improved ventricular contractility, reduced median time to functional recovery (2 days vs 9 days; *p* = 0.02), and lower cardiovascular events (20% vs 79%; *p* < 0.01). To date, after up to 6 years of follow-up since the start of the trial, surviving patients have not presented any significant adverse events attributable to the intervention.

*Lung* Similarly to what is observed for acute cardiac events, a myriad of injurious processes can result in ischemic injuries to the lungs, either by direct pathology (pulmonary embolism, trauma) or by indirect damage (such as cardiopulmonary bypass and resuscitation for cardiac arrest)[1]. Interestingly, the metabolic alterations elicited by ischemia can cause enzymatic repercussions in several pathways, such as nitric oxide synthase, which under anoxia can generate almost exclusively ROS, in detriment of NO [63]. Lung IRI (LIRI) is a complex phenomenon, given the unparalleled scenario of oxygen availability in alveolar spaces even under some conditions of limited perfusion, which is one of the possible manifestations of ventilation-perfusion (V/Q) mismatch [103]. In lung transplantation, for instance, organs are ventilated and harvested under a fraction of inspired oxygen (FiO2) of 50% and kept inflated (ideally) during the entire ischemic time preceding implantation in the recipient. No other organ is subjected to such a unique environment [104]. Moskowitzova et al. showed that mitochondrial transplant could be potentially employed as a treatment option for acute lung injury in a murine model of IRI [105]. By clamping the pulmonary hilum for 2 h, C57BL/6 J mice were randomized to receive autologous mitochondrial transplantation (isolated from the gastrocnemius muscle) or vehicle upon reperfusion, with subsequent evaluation for 24 h. This injury model had previously been shown to induce mitochondrial dysfunction, with decay in complexes I–V, II–V, and III–V, and reduced mitochondrial viability [106]. Both intra-arterial (IA) and nebulized aerosolized mitochondrial transplantation were explored (with dosages of 1 × 10^8^ and 3 × 10^8^ particles, respectively) and compared to vehicle controls [105]. For the unprecedented nebulization group, mitochondria were delivered through an Aeroneb ultrasonic nebulizer over 10 s, followed by 1 min of regular mechanical ventilation, repeated 4 times. Results pointed that mitochondrial transplant, *regardless* of the administration route, significantly improved lung mechanics, with narrower hysteresis and higher compliances, and decreased lung tissue injury under blinded tissue analysis. Transplanted mitochondria were found in the pulmonary artery (IA group) and in the trachea and bronchial three (nebulization group). Additionally, transplanted mitochondria were found in the lung parenchyma of both groups. Strikingly, nearly all evaluated parameters of lung mechanics were not significantly different from non-IRI controls (sham) in either mitochondrial transplant group, as well as being statistically superior to vehicle controls, although no mechanistic studies were explored by the authors.

In addition to acute lung injury caused by warm ischemia, the efficacy of mitochondrial transplant was recently investigated in an extended CIT, lung transplant model with Ex Vivo Lung Perfusion (EVLP) [9]. Since its inception [107], EVLP systems have been clinically employed worldwide by large academic centers in the evaluation of marginal lungs, which would otherwise not be deemed suitable for direct transplantation, with similar long-term outcomes [108]. In this setting, lungs are reperfused under normothermic conditions, mechanically ventilated, and clinically assessed for up to 4 h prior to the decision to proceed or not to transplantation. Cloer et al. explored whether intra-arterial mitochondrial transplant on EVLP resulted in improved functional and molecular outcomes in both a large animal model and in rejected human lungs [9]. In contrast to all previous studies to date, the group opted to apply heterologous mitochondria that had been previously frozen in a trehalose-based buffered solution for up to 2 months.

In a porcine, open-atrium, 6-h EVLP following 22 h of CIT at 4ºC, the authors investigated whether this heterologous mitochondrial transplant by intra-arterial injection at 1 and 4 h of reperfusion (i.e., serial injections at 2 time-points) was superior to vehicle controls [9]. Functional assessments indicated that mitochondrial transplant significantly decreased pulmonary vascular resistance as compared to vehicle controls, when normalized to baseline. No differences were observed in oxygenation capacity as measured by partial pressure of arterial oxygen over fraction of inspired oxygen (P/F ratio), and no other measures of lung physiology were reported. Interestingly, the same functional findings were encountered when performing a similar experiment with clinically rejected human lungs. For the human study, lungs were deemed eligible when total CIT was less than 30 h, with subsequent analysis for 4 h on EVLP. Mitochondrial transplant or vehicle (*n* = 5/group) was then administered at 2 h of reperfusion, and a second injection was given when flushing the lungs with the standard low-potassium dextran solution at the end of EVLP, preceding a second CIT of up to 18 h. This design was described as mimicking an extended shipment of lung tissue from the EVLP site to a potential clinic.

From the analysis of EVLP tissue lysate samples and single cell suspension studies modelled to simulate cellular behavior during ex-vivo reperfusion, Cloer et al. [9] demonstrated that mitochondrial transplant reduced inflammatory response and improved antioxidant capacity. Furthermore, mitochondrial transplant reduced cell death by over 30% and increased live cell count by 25% when compared to controls, which was reinforced by marked reduction of phospho-mixed lineage kinase domain-like protein (pMLKL). In support of recent studies, no oxidative or proinflammatory response was observed following injection of a porcine-derived xenogeneic mitochondrial transplant in human tissue. This important xenogeneic experiment also provided additional insights for the future translation of non-autologous mitochondrial transplant. In an additional exploratory in-vitro experiment, human pulmonary artery endothelial cells (HPAEC) exposed to simulated CIT at 4 °C for 24 h were treated with either exogenous porcine mitochondria (at 100 to 1000 particles/cell) or vehicle upon rewarming. Surprisingly, benefits from mitochondrial co-culture (increased viability and autophagy, and decreased CXCL8, MCP1, and 8-OHdG) were sustained for the entire culture period of 7 days, despite a relatively expeditious drop of mRNA expression of porcine ssMtND5. This finding is encouraging from a safety translation standpoint and suggests the efficacy of mitochondrial transplant even with xenogeneic sources.

*Liver* In rodent models of acute liver injury, induced either by IRI or acetaminophen (APAP), mitochondrial transplantation has been shown to be safe and efficacious [109–112]. In rat liver IRI, Ko et al. [110] and Lin et al. [111] have independently demonstrated that intrasplenic injection of mitochondria or melatonin-pretreated mitochondria markedly reduced liver enzyme release, oxidative stress, and hepatocellular injury. In addition, Ko et al. [110] demonstrated that melatonin-pretreated mitochondrial transplantation reduced circulating pro-inflammatory cytokines, such as IL-6, TNFα and MPO, while improving liver ATP and NADH content. Moreover, liver tissue ETC components–complexes I, II, III and V–increased in protein expression in response to mitochondrial transplantation, as compared to I/R-only control rats. This suggested a restoration in the mitochondrial integrity and oxidative phosphorylation in treated rats. Finally, Ko et al. [110] demonstrated that the infiltration of CD68+ and CD14+ cells was reduced in melatonin-pretreated mitochondrial transplantation in the setting of liver IRI, suggesting further the nullification of the early cellular inflammatory response.

Another possible therapeutic avenue is the use of pre-ischemic conditioning prior to liver transplantation and ex vivo liver perfusion. Exploring the use of pre-ischemic mitochondrial transplantation has potential to improve the function of live donor livers destined for clinical transplant, which inevitably will be exposed to a WIT. Guariento et al. [99] demonstrated that pre-ischemic mitochondrial transplantation in porcine hearts significantly improved resilience to IRI and mitigated the detrimental effects of the ischemic event. Considering the potential efficacy of mitochondrial transplantation in cardiac model systems, here, we infer possible uses of this novel therapeutic in liver transplantation and elective liver surgery.

Similarly, in mouse models of APAP-induced acute liver toxicity, mitochondrial transplantation was able to increase hepatocyte ATP content, reduce oxidative stress, while decreasing hepatocellular injury, although no clear mechanism was elucidated [109, 112]. Identification of molecular signaling pathways and cellular mechanisms are required for further understanding of how mitochondrial transplantation confers its hepatoprotective capacity.

*Brain* Ischemic brain injuries, which can be consequence of obstruction of blood flow to the brain due to intravascular thrombi, embolization, hypotension, or extrinsic vessel compression following head trauma, lead to a cellular energy crisis alongside oxidative stress, inflammation, apoptosis, and mitochondrial dysfunction [113]. This is due to the oxygen and glucose deprivation to the neurons impacting ATP production and promoting glycolytic metabolism that increases lactate production [114]. Such injuries can lead to neuronal apoptosis and astrogliosis affecting cognitive and motor functions. Current treatments that address mitochondrial dysfunction include hyperbaric oxygen, exercise, and antioxidant therapy [115]. However, noting the central role of the mitochondria in these injuries suggests the potential of introducing healthy mitochondria as a form of treatment [116].

In recent years, several in vivo experiments have explored the benefits of mitochondrial transplantation in treating brain ischemia. Models of injury on mice and rat include controlled cortical impact (CCI) for traumatic brain injury (TBI) [115, 117] and middle cerebral artery occlusion (MCAO) for cerebral ischemia [116]. Following injury, intracerebroventricular injections (ICV) were used to perform mitochondrial transplantation [115–117]. Different studies have used various sources of mitochondria including mesenchymal stem cells, allogeneic liver and muscle biopsies, or autologous muscle biopsies and have all yielded promising evidence supporting the benefits of mitochondrial transplantation [115–117].

Studies have demonstrated that mitochondrial transplantation in a TBI mouse model lowered blood brain barrier (BBB) damage and brain water content, improved OCR, and promoted angiogenesis [115]. Additionally, mitochondrial transplant promoted expression of brain derived neurotrophic factor (BDNF), which is important in supporting neuron survival, including both astrocytes and microglia [117]. Spatial memory and cognitive function were also improved in mice with mitochondrial transplantation [117]. These results support the importance of replenishing mitochondria following brain IRI and the central role mitochondria play in the mechanism of brain injury.

Ischemic injury promotes astrogliosis, which is a critical factor for hindering regeneration in response to neural injury [118]. This is linked to cellular energy disruption and oxidative stress and thus neuronal loss in response to decreased energy to meet demands [114]. Mitochondrial transplant in a rat cerebral IRI model reduced astrogliosis in the mitochondrial transplant group compared to the vehicle control [118]. Furthermore, reduced levels of the ischemia biomarker, CPK, and decreased infarct area were also measured following mitochondria transplant [119]. Moreover, mitochondrial transplants have also been shown to significantly reduce IRI-induced apoptosis in brain cells [119, 120] and reduce the volume of infarcted tissue [121]. Currently, a clinical trial led by Dr. Melanie Walker at the University of Washington is investigating the utility of mitochondrial transplantation on cerebral ischemia (NCT04998357, recruiting patients) [6].

*Kidney* Several studies utilizing acute kidney injury in rats have demonstrated therapeutic benefit of mitochondrial transplant [122–125]. Doulamis et al. [122] demonstrated that intra-arterial delivery of donor mitochondria significantly improved kidney function following IRI in a porcine model. Specifically, mitochondrial transplant provided renal protection against IRI-induced decreases in glomerular filtration rate and urine output as well as provided partial protection against tissue necrosis [122]. These findings were confirmed by Rossi et al. [123], which demonstrated improved tissue health and function. Mitochondrial transplant is currently being explored by Cellvie Inc. as a therapeutic approach to improve tissue health prior to kidney transplantation.

### Regulatory considerations

As mitochondrial transplantation is emerging as a new therapeutic, there has been extensive consultation with regulators, particular the Food and Drug Administration (FDA) on the clinical trial design considerations, particularly in applications for primary mitochondrial diseases; these have included several workshops [126–129]. These workshops have neatly outlined the challenges of capturing significant effects in relatively rare primary mitochondrial disease patient populations. Clinical evaluation of mitochondrial transplantation in cerebral ischemia and in patients on ECMO for postcardiotomy cardiogenic shock have used autologous muscle-tissue derived mitochondria with little information published on the quality (viability, potency) and quantity (2 × 10^10^ viable and respiration competent mitochondria); the focus has appropriately been on feasibility and safety. Analogously, cell and gene modified cell therapies have evolved over many decades to relatively harmonized global regulatory requirements which account for multiple safety (free from adventitious agents), and quality metrics (viability, dose, multivariate potency readouts reflective of clinically relevant mechanism of action) before such investigational products can be used in clinical trial investigations. Thus, the field of mitochondrial transplantation will similarly evolve to consider quality metrics such as the purity, viability, quantity and potency of isolated mitochondria; the original cell sources will have to be appropriately screened for adventitious agents. Understanding mitochondrial transplant mechanism of action, effects on mtDNA copy numbers and variants produced and extent of uptake will inform the type of quality control metrics that regulators will require as clinical trials advance beyond initial feasibility and safety phases.

### Perspectives

The examination of the literature on the efficacy of mitochondrial transplantation for acute diseases provides some encouraging findings. Primarily, for IRI in diverse organ systems, it is possible to observe not only a favorable molecular, chemical, and immunological modulation, but also *functional* benefits in several animal models. Although most of the data only includes short periods of functional evaluation of target subjects, it is noteworthy that mitochondrial transplantation seems to provide *early*, measurable physiological improvement in target tissues. Additionally, IRI models suggest that mitochondrial transplantation can also significantly reduce tissue infarction, apoptosis, and necrosis, therefore presenting implications on tissue viability and potential for organ regeneration. Of note, published data also supports the hypothesis that transplanted mitochondria may be retained in tissue longer-term, for at least 4 weeks in porcine and rabbit survival models, while maintaining the benefits of reduced infarct size area [130, 131].

With regards to safety, some interesting observations can be drawn from animal and human studies. Published clinical data on mitochondrial transplantation is derived from a single center and is limited to an *autologous* mitochondrial isolation technique, but with a relatively extended follow-up (now surpassing 6 years for some patients), and no observable adverse events have been identified [102]. Several animal models support the hypothesis that *heterologous* mitochondrial transplantation may not elicit hyperacute, acute, or chronic rejection, and no increase in pro-inflammatory chemokines and cytokines. Porcine-derived fibroblasts providing heterologous mitochondria to porcine hearts, porcine left-ventricle-derived heterologous mitochondria to porcine lungs, or murine skeletal-muscle-derived heterologous mitochondria to murine hearts are examples of intra-species, heterologous mitochondrial transplantation studies from which those observations can be drawn [9, 96, 99, 100]. Importantly, Ramirez-Barbieri et al. [97] did not identify direct or indirect, acute or chronic alloreactivity, allorecognition or DAMPs to mitochondrial transplantation when directly comparing syngeneic and allogeneic (heterologous) mitochondria. *Xenogeneic* (interspecies) mitochondrial transplantation models, either assessing mitochondrial uptake [130] or efficacy [9], also did not encounter any detrimental effects on target organs or organisms, with increasingly abundant data from animal models, although data on human tissue uptake is still sparse [9]. From a translational standpoint, however, autologous mitochondrial transplantation is naturally the safest route for initial studies, which poses implications on the disease models for which this therapy could be potentially first explored. The possibility of storing well-preserved, functional mitochondria needs to be further replicated by other centers before further conclusions can be drawn but could significantly impact the applicability and generalizability of mitochondrial transplantation [9]. For instance, organ transplant models that evaluate extended CIT are currently largely prevented from using autologous mitochondrial transplantation owing to the logistic limitations of limited mitochondrial viability in respiration buffers.

### Limitations and unanswered questions

Many are the questions that remain to be elucidated. From a study design perspective, it is still unclear, for instance, if single or serial mitochondrial transplants should be prioritized for initial translational studies. Given the variable impact of serial mitochondrial applications [96, 98]—which is likely influenced by metabolic demands that are dependent both of target tissue, intraspecies (age effect) and interspecies model limitations (smaller animals with higher metabolic demands seem to require proportionally higher mitochondrial concentrations)—and given safety should be the primary concern, it seems reasonable to utilize serial injections first. It is also unclear whether the source of mitochondria should be of concern. Most published data support the use of skeletal-muscle or myocardium as sources of mitochondria for several organs, and although claims have been made in support of equivalence regardless of the donor tissue, data is limited and conflicting. Zhang et al. recently showed that despite cell origin not impacting on the overall benefit of mitochondrial transplantation for rescuing apoptosis from doxorubicin-induced heart failure, metabolically matched mitochondria were necessary for improving contractile function [50]. Masuzawa et al. [131], however, indicated that for acute ischemic heart events, liver-derived isolated mitochondria are as effective as skeletal-muscle-derived mitochondria.

From a mechanistic perspective, several key aspects remain unanswered, such as the exact therapeutic mechanism, the potential of biomaterial coatings to enhance therapeutic efficacy, and the ability to store healthy donor mitochondria. Further studies are needed to investigate how donor mitochondria elicit a therapeutic benefit following engulfment by recipient cells. Preclinical animal models found that only 44% of donor mitochondria were colocalized with cardiomyocytes, with the remainder in other cell types and interstitial space [130, 132]. The use of biomaterials to coat donor mitochondria is important therapeutically to understand if it is possible to further enhance cellular engulfment of donor mitochondria and target specific cell types within a tissue to improve therapeutic efficacy. Biomaterial coatings may also be beneficial in stabilizing isolated mitochondria, which have a short window of viability that hinders clinical utilization. The lack of ability to store isolated mitochondria to perform necessary quality control is a significant limitation to the clinical advancement of mitochondrial transplant. Currently, mitochondrial isolation must occur immediately prior to transplant into the patient with minimal time to perform assays to ensure mitochondrial purity and viability. The potential of storing isolated mitochondria in trehalose buffers [9] or other solutions represent a significant advancement in the mitochondrial transplant field. This would allow the creation of mitochondrial banks with fully characterized mitochondria to maximize patient safety and therapeutic benefit. Overall, the mitochondrial transplant field is still developing, and several challenges remain prior to full clinical utilization.

## Conclusions

**Fig. 2:**
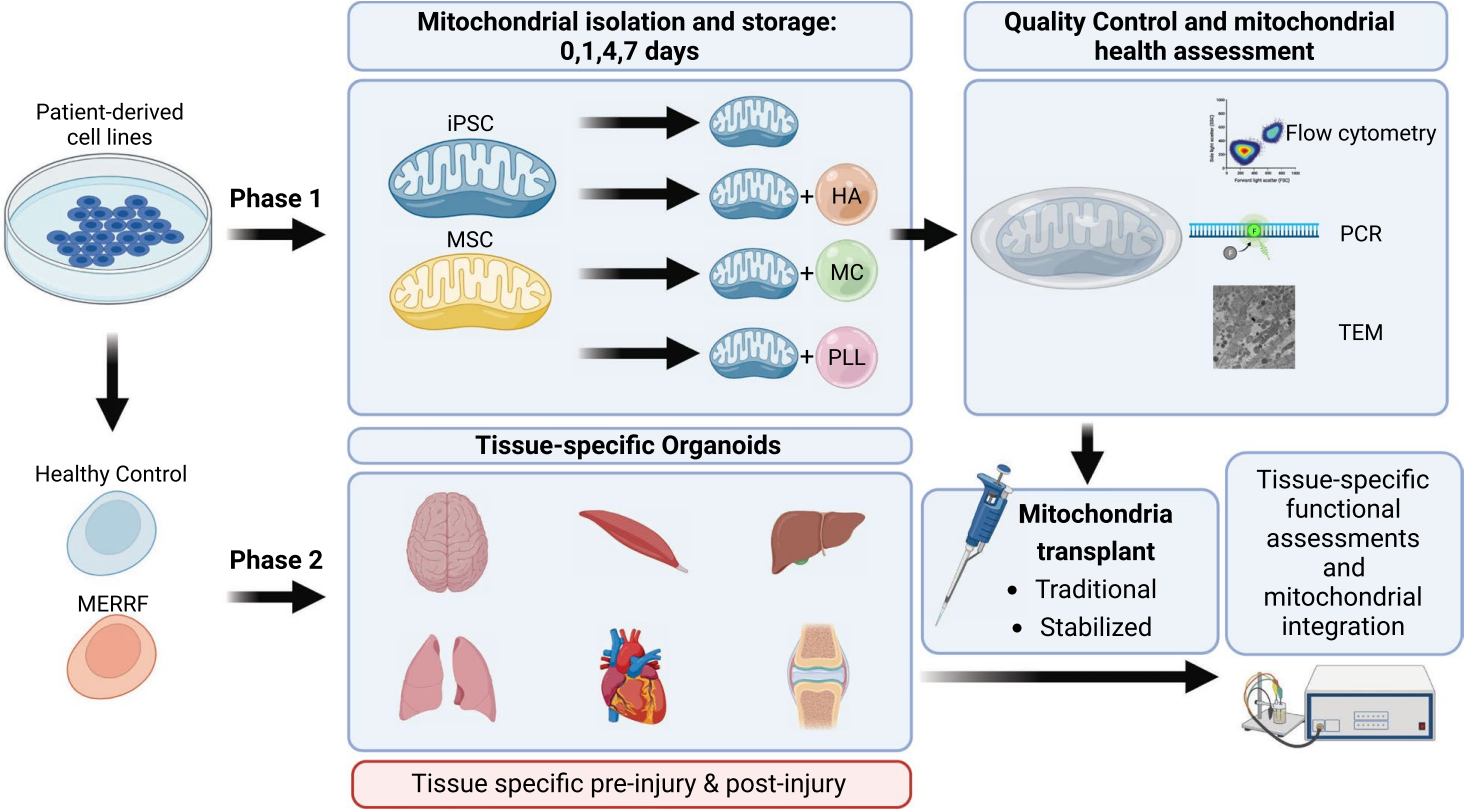
Our mitochondrial transplant CWG research plan to investigate the stabilization of isolated mitochondria and therapeutic efficacy in organ-on-a-chip models. Figure created with BioRender.com

Overall, the therapeutic efficacy of mitochondria transplant is promising in animal and early clinical trials, but many questions remain. Our mitochondrial transplant CWG has identified priorities to accelerate the pace of translational progress including the need for improved storage and enhanced delivery of isolated mitochondria, along with the development of accurate in vitro models to recapitulate the complexity of human tissue. Our interdisciplinary team developed a research plan (Fig. 2) to evaluate the stabilization and short-term storage of isolated mitochondria encapsulated in hyaluronic acid, methyl cellulose, and poly(L-lysine). The ability to store isolated mitochondria prior to transplantation will enable clinicians to perform comprehensive quality control assessments, ensuring that only healthy and pure mitochondria are transplanted, potentially reducing the risk of adverse events. Stored mitochondria can also be utilized in procedures requiring multiple mitochondrial transplants, such as prior to organ harvesting and donation, without requiring repeated mitochondrial isolations. We will also utilize brain, cardiac, muscle, joint, lung, and liver organ-on-a-chip models to evaluate mitochondrial transplant in acute and chronic diseases states. This will allow us to accurately assess the clinical applicability of the mitochondrial transplant in vitro using human-derived samples and investigate the therapeutic mechanisms. Advancements in these areas are important for the clinical applicability and translation of mitochondrial transplants in a range of diseases.

## Data Availability

Not applicable.

## Abbreviations

IRI: Ischemia–reperfusion injury
MITO2i: Mitochondrial Innovation Initiative
MbD: Medicine by design
CWG: Convergence working group
mtDNA: Mitochondrial DNA
ETC: Electron transport chain
nDNA: Nuclear DNA
ECMO: Extracorporeal membrane oxygenation
HSPC: Hematopoietic stem and progenitor cells
Dextran-TPP: Triphenylphosphonium-conjugated Dextran
PEO98-PPO67-PEO98: Poly(ethylene oxide)98-poly(propylene oxide)67-poly(ethylene oxide)98
HA: Hyaluronic acid
MC: Methylcellulose
OCR: Oxygen consumption rate
DOTAP: Dioleoyl-3-trimethylammonium propane
DOPE: 1,2-Dioleoyl-sn-glycero-3-phosphoethanolamine
PEG: Poly(ethylene glycol)
GOx: Glucose oxidase
DAMP: Damage associated molecular patterns
MP: Transmembrane potential
mPTP: Mitochondrial permeability transition pore
dsDNA: Double strand DNA
ROS: Reactive oxygen species
RNS: Reactive nitrogen species
CIT: Cold ischemic time
WIT: Warm ischemia time
DCD: Donation after circulatory death
OoC: Organ-on-a-chip
JoC: Joint-on-a-chip
EVHP: Ex vivo heart perfusion
LAD: Left anterior descending artery
LIRI: Lung ischemia–reperfusion injury
V/Q: Ventilation-perfusion
FiO2: Fraction of inspired oxygen
IA: Intra-arterial
EVLP: Ex vivo lung perfusion
P/F: Partial pressure of arterial oxygen over fraction of inspired oxygen
pMLKL: Phospho-mixed lineage kinase domain-like protein
HPAEC: Human Pulmonary Artery Endothelial Cells
APAP: Acetaminophen
CCI: Controlled cortical impact
TBI: Traumatic brain injury
MCAO: Middle cerebral artery occlusion
ICV: Intracerebroventricular injections
BBB: Blood brain barrier
BDNF: Brain derived neurotrophic factor

## References

[1] D'Amato M, Morra F, Di Meo I, Tiranti V (2023). Mitochondrial transplantation in mitochondrial medicine: current challenges and future perspectives. Int J Mol Sci..

[2] Gorman GS, Chinnery PF, DiMauro S, Hirano M, Koga Y, McFarland R (2016). Mitochondrial diseases. Nat Rev Dis Primers.

[3] Wiedemann N, Pfanner N (2017). Mitochondrial machineries for protein import and assembly. Annu Rev Biochem.

[4] Latorre-Pellicer A, Moreno-Loshuertos R, Lechuga-Vieco AV, Sánchez-Cabo F, Torroja C, Acín-Pérez R (2016). Mitochondrial and nuclear DNA matching shapes metabolism and healthy ageing. Nature.

[5] Emani SM, Piekarski BL, Harrild D, Del Nido PJ, McCully JD (2017). Autologous mitochondrial transplantation for dysfunction after ischemia–reperfusion injury. J Thorac Cardiovasc Surg.

[6] Walker M. Autologous Mitochondrial Transplant for Cerebral Ischemia. Autologous Mitochondrial Transplant for Cerebral Ischemia: https://www.clinicaltrials.gov/study/NCT04998357.

[7] Kesner EE, Saada-Reich A, Lorberboum-Galski H (2016). Characteristics of mitochondrial transformation into human cells. Sci Rep.

[8] Pacak CA, Preble JM, Kondo H, Seibel P, Levitsky S, Del Nido PJ (2015). Actin-dependent mitochondrial internalization in cardiomyocytes: evidence for rescue of mitochondrial function. Biol Open.

[9] Cloer CM, Givens CS, Buie LK, Rochelle LK, Lin YT, Popa S (2023). Mitochondrial transplant after ischemia reperfusion promotes cellular salvage and improves lung function during ex-vivo lung perfusion. J Heart Lung Transplant.

[10] Davis CH, Kim KY, Bushong EA, Mills EA, Boassa D, Shih T (2014). Transcellular degradation of axonal mitochondria. Proc Natl Acad Sci USA.

[11] Liu D, Gao Y, Liu J, Huang Y, Yin J, Feng Y (2021). Intercellular mitochondrial transfer as a means of tissue revitalization. Signal Transduct Target Ther.

[12] Pang Y, Zhang C, Gao J (2021). Macrophages as emerging key players in mitochondrial transfers. Front Cell Dev Biol.

[13] Jacoby E, Ben Yakir-Blumkin M, Blumenfeld-Kan S, Brody Y, Meir A, Melamed-Book N (2021). Mitochondrial augmentation of CD34. NPJ Regen Med.

[14] Maeda H, Kami D, Maeda R, Shikuma A, Gojo S (2021). Generation of somatic mitochondrial DNA-replaced cells for mitochondrial dysfunction treatment. Sci Rep.

[15] Morrison TJ, Jackson MV, Cunningham EK, Kissenpfennig A, McAuley DF, O'Kane CM (2017). Mesenchymal stromal cells modulate macrophages in clinically relevant lung injury models by extracellular vesicle mitochondrial transfer. Am J Respir Crit Care Med.

[16] Mukkala AN, Jerkic M, Khan Z, Szaszi K, Kapus A, Rotstein O (2023). Therapeutic effects of mesenchymal stromal cells require mitochondrial transfer and quality control. Int J Mol Sci..

[17] Hubbard WB, Harwood CL, Prajapati P, Springer JE, Saatman KE, Sullivan PG (2019). Fractionated mitochondrial magnetic separation for isolation of synaptic mitochondria from brain tissue. Sci Rep.

[18] Preble JM, Pacak CA, Kondo H, MacKay AA, Cowan DB, McCully JD (2014). Rapid isolation and purification of mitochondria for transplantation by tissue dissociation and differential filtration. J Vis Exp.

[19] Mitchell MJ, Billingsley MM, Haley RM, Wechsler ME, Peppas NA, Langer R (2021). Engineering precision nanoparticles for drug delivery. Nat Rev Drug Discov.

[20] Bernhard S, Tibbitt MW (2021). Supramolecular engineering of hydrogels for drug delivery. Adv Drug Deliv Rev.

[21] Souery WN, Bishop CJ (2018). Clinically advancing and promising polymer-based therapeutics. Acta Biomater.

[22] Khaliq NU, Lee J, Kim S, Sung D, Kim H (2023). Pluronic F-68 and F-127 based nanomedicines for advancing combination cancer therapy. Pharmaceutics.

[23] Bray D, Hopkins C, Roberts DN (2010). A review of dermal fillers in facial plastic surgery. Curr Opin Otolaryngol Head Neck Surg.

[24] Alconcel SNS, Baas AS, Maynard HD (2011). FDA-approved poly(ethylene glycol)–protein conjugate drugs. Polym Chem.

[25] Abourehab MAS, Pramanik S, Abdelgawad MA, Abualsoud BM, Kadi A, Ansari MJ (2022). Recent advances of chitosan formulations in biomedical applications. Int J Mol Sci..

[26] Burdock GA (2007). Safety assessment of hydroxypropyl methylcellulose as a food ingredient. Food Chem Toxicol.

[27] Liu J, Liu B (2023). Material-assisted engineering of organelles for biomedical applications. ACS Mater Lett.

[28] Wu S, Zhang A, Li S, Chatterjee S, Qi R, Segura-Ibarra V (2018). Polymer functionalization of isolated mitochondria for cellular transplantation and metabolic phenotype alteration. Adv Sci.

[29] Liu H, Wu S, Lee H, Baudo G, Massaro M, Zhang A (2022). Polymer-functionalized mitochondrial transplantation to plaque macrophages as a therapeutic strategy targeting atherosclerosis. Adv Ther..

[30] Alexander J, Mahalingam R, Seua A, Wu S, Arroyo L, Horbelt T (2022). Targeting the meningeal compartment to resolve chemobrain and neuropathy via nasal delivery of functionalized mitochondria. Adv Healthc Mater.

[31] Biswas S, Dodwadkar N, Piroyan A, Torchilin V (2012). Surface conjugation of triphenylphosphonium to target poly(amidoamine) dendrimers to mitochondria. Biomaterials.

[32] Huang Y, Sun X, Gao R, Zhang L, Chen H, Lv Y (2023). Transplantation of mitochondria encapsulated in hydrogel ameliorates myocardial ischemia–reperfusion injury. Chem Eng J..

[33] Patel S, Michael F, Khan M, Duggan B, Wyse S, Darby D (2022). Erodible thermogelling hydrogels for localized mitochondrial transplantation to the spinal cord. Mitochondrion.

[34] Chen W, Shi K, Chu B, Wei X, Qian Z (2019). Mitochondrial surface engineering for multidrug resistance reversal. Nano Lett.

[35] Nakano T, Nakamura Y, Park JH, Tanaka M, Hayakawa K (2022). Mitochondrial surface coating with artificial lipid membrane improves the transfer efficacy. Commun Biol.

[36] Chen W, Huang T, Shi K, Chu B, Qian Z (2020). Chemotaxis-based self-accumulation of surface-engineered mitochondria for cancer therapeutic improvement. Nano Today.

[37] Yu X, Lyu M, Ou X, Liu W, Yang X, Ma X (2023). AIEgens/mitochondria nanohybrids as bioactive microwave sensitizers for non-thermal microwave cancer therapy. Adv Healthc Mater.

[38] Liu J, Liu X, Wu M, Qi G, Liu B (2020). Engineering living mitochondria with AIE photosensitizer for synergistic cancer cell ablation. Nano Lett.

[39] Doulamis IP, McCully JD (2021). Mitochondrial transplantation for ischemia reperfusion injury. Methods Mol Biol.

[40] Grazioli S, Pugin J (2018). Mitochondrial damage-associated molecular patterns: from inflammatory signaling to human diseases. Front Immunol.

[41] McCully JD, Cowan DB, Pacak CA, Toumpoulis IK, Dayalan H, Levitsky S (2009). Injection of isolated mitochondria during early reperfusion for cardioprotection. Am J Physiol Heart Circ Physiol.

[42] Yamaguchi R, Andreyev A, Murphy AN, Perkins GA, Ellisman MH, Newmeyer DD (2007). Mitochondria frozen with trehalose retain a number of biological functions and preserve outer membrane integrity. Cell Death Differ.

[43] Bodenstein D, Kim H, Brown N, Navaid B, Young L, Andreazza A (2019). Mitochondrial DNA content and oxidation in bipolar disorder and its role across brain regions. NPJ Schizophr.

[44] Venegas V, Halberg MC, Wong PDL (2012). Measurement of mitochondrial DNA copy number. Mitochondrial disorders: biochemical and molecular analysis.

[45] Popov LD (2020). Mitochondrial biogenesis: an update. J Cell Mol Med.

[46] Yang M, Linn BS, Zhang Y, Ren J (2019). Mitophagy and mitochondrial integrity in cardiac ischemia–reperfusion injury. Biochim Biophys Acta Mol Basis Dis.

[47] Preble JM, Kondo H, Levitsky S, McCully JD (2013). Quality control parameters for mitochondria transplant in cardiac tissue. Mol Biol.

[48] Fernández-Vizarra E, Ferrín G, Pérez-Martos A, Fernández-Silva P, Zeviani M, Enríquez JA (2010). Isolation of mitochondria for biogenetical studies: an update. Mitochondrion.

[49] Cadoná FC, de Souza DV, Fontana T, Bodenstein DF, Ramos AP, Sagrillo MR (2021). Açaí (Euterpe oleracea Mart.) as a potential anti-neuroinflammatory Agent: NLRP3 priming and activating signal pathway modulation. Mol Neurobiol..

[50] Zhang A, Liu Y, Pan J, Pontanari F, Chia-Hao Chang A, Wang H (2023). Delivery of mitochondria confers cardioprotection through mitochondria replenishment and metabolic compliance. Mol Ther.

[51] Shin B, Saeed MY, Esch JJ, Guariento A, Blitzer D, Moskowitzova K (2019). A novel biological strategy for myocardial protection by intracoronary delivery of mitochondria: safety and efficacy. JACC Basic Transl Sci.

[52] Varnamkhasti BS, Hosseinzadeh H, Azhdarzadeh M, Vafaei SY, Esfandyari-Manesh M, Mirzaie ZH (2015). Protein corona hampers targeting potential of MUC1 aptamer functionalized SN-38 core-shell nanoparticles. Int J Pharm.

[53] Deng ZJ, Liang M, Toth I, Monteiro MJ, Minchin RF (2012). Molecular interaction of poly(acrylic acid) gold nanoparticles with human fibrinogen. ACS Nano.

[54] Bertrand N, Grenier P, Mahmoudi M, Lima EM, Appel EA, Dormont F (2017). Mechanistic understanding of in vivo protein corona formation on polymeric nanoparticles and impact on pharmacokinetics. Nat Commun.

[55] Ke PC, Lin S, Parak WJ, Davis TP, Caruso F (2017). A decade of the protein corona. ACS Nano.

[56] Dai Q, Bertleff-Zieschang N, Braunger JA, Björnmalm M, Cortez-Jugo C, Caruso F (2018). Particle targeting in complex biological media. Adv Healthc Mater..

[57] Nel AE, Mädler L, Velegol D, Xia T, Hoek EM, Somasundaran P (2009). Understanding biophysicochemical interactions at the nano-bio interface. Nat Mater.

[58] Ali A, Wang A, Ribeiro RVP, Beroncal EL, Baciu C, Galasso M (2021). Static lung storage at 10°C maintains mitochondrial health and preserves donor organ function. Sci Transl Med..

[59] Saeb-Parsy K, Martin JL, Summers DM, Watson CJE, Krieg T, Murphy MP (2021). Mitochondria as therapeutic targets in transplantation. Trends Mol Med.

[60] Amo T, Yadava N, Oh R, Nicholls DG, Brand MD (2008). Experimental assessment of bioenergetic differences caused by the common European mitochondrial DNA haplogroups H and T. Gene.

[61] Marcuello A, Martínez-Redondo D, Dahmani Y, Casajús JA, Ruiz-Pesini E, Montoya J (2009). Human mitochondrial variants influence on oxygen consumption. Mitochondrion.

[62] Jiménez-Sousa MA, Tamayo E, Guzmán-Fulgencio M, Fernández-Rodríguez A, Heredia-Rodriguez M, García-Álvarez M (2014). Relationship between European mitochondrial haplogroups and chronic renal allograft rejection in patients with kidney transplant. Int J Med Sci.

[63] den Hengst WA, Gielis JF, Lin JY, Van Schil PE, De Windt LJ, Moens AL (2010). Lung ischemia–reperfusion injury: a molecular and clinical view on a complex pathophysiological process. Am J Physiol Heart Circ Physiol.

[64] Boyle EM, Canty TG, Morgan EN, Yun W, Pohlman TH, Verrier ED (1999). Treating myocardial ischemia–reperfusion injury by targeting endothelial cell transcription. Ann Thorac Surg.

[65] Hayashida K, Takegawa R, Shoaib M, Aoki T, Choudhary RC, Kuschner CE (2021). Mitochondrial transplantation therapy for ischemia reperfusion injury: a systematic review of animal and human studies. J Transl Med.

[66] Fischer S, Maclean AA, Liu M, Cardella JA, Slutsky AS, Suga M (2000). Dynamic changes in apoptotic and necrotic cell death correlate with severity of ischemia–reperfusion injury in lung transplantation. Am J Respir Crit Care Med.

[67] Iskender I, Cypel M, Martinu T, Chen M, Sakamoto J, Kim H (2018). Effects of warm versus cold ischemic donor lung preservation on the underlying mechanisms of injuries during ischemia and reperfusion. Transplantation.

[68] Belzer FO, Southard JH (1988). Principles of solid-organ preservation by cold storage. Transplantation.

[69] Russo MJ, Iribarne A, Hong KN, Ramlawi B, Chen JM, Takayama H (2010). Factors associated with primary graft failure after heart transplantation. Transplantation.

[70] Halazun KJ, Al-Mukhtar A, Aldouri A, Willis S, Ahmad N (2007). Warm ischemia in transplantation: search for a consensus definition. Transplant Proc.

[71] Kwon JH, Blanding WM, Shorbaji K, Scalea JR, Gibney BC, Baliga PK (2023). Waitlist and transplant outcomes in organ donation after circulatory death: trends in the United States. Ann Surg..

[72] Sánchez-Cámara S, Asensio-López MC, Royo-Villanova M, Soler F, Jara-Rubio R, Garrido-Peñalver JF (2022). Critical warm ischemia time point for cardiac donation after circulatory death. Am J Transplant.

[73] Musso V, Righi I, Damarco F, Mazzucco A, Zanella A, Vivona L (2021). Lung donation after circulatory death. Curr Chall Thor Surg..

[74] Duong A, Evstratova A, Sivitilli A, Hernandez JJ, Gosio J, Wahedi A (2021). Characterization of mitochondrial health from human peripheral blood mononuclear cells to cerebral organoids derived from induced pluripotent stem cells. Sci Rep.

[75] Zhao Y, Wang EY, Lai FBL, Cheung K, Radisic M (2023). Organs-on-a-chip: a union of tissue engineering and microfabrication. Trends Biotechnol.

[76] Chal J, Al Tanoury Z, Hestin M, Gobert B, Aivio S, Hick A (2016). Generation of human muscle fibers and satellite-like cells from human pluripotent stem cells in vitro. Nat Protoc.

[77] Zhao Y, Rafatian N, Feric NT, Cox BJ, Aschar-Sobbi R, Wang EY (2019). A platform for generation of chamber-specific cardiac tissues and disease modeling. Cell..

[78] Sivitilli AA, Gosio JT, Ghoshal B, Evstratova A, Trcka D, Ghiasi P (2020). Robust production of uniform human cerebral organoids from pluripotent stem cells. Life Sci Alliance..

[79] Zhao Y, Rafatian N, Feric NT, Cox BJ, Aschar-Sobbi R, Wang EY (2019). A platform for generation of chamber-specific cardiac tissues and disease modeling. Cell.

[80] Lust ST, Shanahan CM, Shipley RJ, Lamata P, Gentleman E (2021). Design considerations for engineering 3D models to study vascular pathologies in vitro. Acta Biomater.

[81] Zhang J, Nuebel E, Wisidagama DR, Setoguchi K, Hong JS, Van Horn CM (2012). Measuring energy metabolism in cultured cells, including human pluripotent stem cells and differentiated cells. Nat Protoc.

[82] Bosakova V, De Zuani M, Sladkova L, Garlikova Z, Jose SS, Zelante T (2022). Lung organoids—The ultimate tool to dissect pulmonary diseases?. Front Cell Dev Biol.

[83] Zhang B, Montgomery M, Chamberlain MD, Ogawa S, Korolj A, Pahnke A (2016). Biodegradable scaffold with built-in vasculature for organ-on-a-chip engineering and direct surgical anastomosis. Nat Mater.

[84] Ronaldson-Bouchard K, Ma SP, Yeager K, Chen T, Song L, Sirabella D (2018). Advanced maturation of human cardiac tissue grown from pluripotent stem cells. Nature.

[85] Feyen DAM, McKeithan WL, Bruyneel AAN, Spiering S, Hormann L, Ulmer B (2020). Metabolic maturation media improve physiological function of human iPSC-derived cardiomyocytes. Cell Rep.

[86] Tomaskovic-Crook E, Zhang P, Ahtiainen A, Kaisvuo H, Lee CY, Beirne S (2019). Human neural tissues from neural stem cells using conductive biogel and printed polymer microelectrode arrays for 3D electrical stimulation. Adv Healthc Mater.

[87] Wikswo JP, Block FE, Cliffel DE, Goodwin CR, Marasco CC, Markov DA (2013). Engineering challenges for instrumenting and controlling integrated organ-on-chip systems. IEEE Trans Biomed Eng.

[88] Chen T, Vunjak-Novakovic G (2019). Human tissue-engineered model of myocardial ischemia–reperfusion injury. Tissue Eng Part A.

[89] Davies LC, Jenkins SJ, Allen JE, Taylor PR (2013). Tissue-resident macrophages. Nat Immunol.

[90] Vormann MK, Tool LM, Ohbuchi M, Gijzen L, van Vught R, Hankemeier T (2022). Modelling and prevention of acute kidney injury through ischemia and reperfusion in a combined human renal proximal tubule/blood vessel-on-a-chip. Kidney630.

[91] Wevers NR, Nair AL, Fowke TM, Pontier M, Kasi DG, Spijkers XM (2021). Modeling ischemic stroke in a triculture neurovascular unit on-a-chip. Fluids Barriers CNS.

[92] Yadid M, Lind JU, Ardona HAM, Sheehy SP, Dickinson LE, Eweje F (2020). Endothelial extracellular vesicles contain protective proteins and rescue ischemia–reperfusion injury in a human heart-on-chip. Sci Transl Med..

[93] Makarczyk MJ, Gao Q, He Y, Li Z, Gold MS, Hochberg MC (2021). Current models for development of disease-modifying osteoarthritis drugs. Tissue Eng Part C Methods.

[94] Samvelyan HJ, Hughes D, Stevens C, Staines KA (2021). Models of osteoarthritis: relevance and new insights. Calcif Tissue Int.

[95] Banh L, Cheung KK, Chan MWY, Young EWK, Viswanathan S (2022). Advances in organ-on-a-chip systems for modelling joint tissue and osteoarthritic diseases. Osteoarthr Cartil.

[96] Guariento A, Doulamis IP, Duignan T, Kido T, Regan WL, Saeed MY (2020). Mitochondrial transplantation for myocardial protection in ex-situ-perfused hearts donated after circulatory death. J Heart Lung Transplant.

[97] Ramirez-Barbieri G, Moskowitzova K, Shin B, Blitzer D, Orfany A, Guariento A (2019). Alloreactivity and allorecognition of syngeneic and allogeneic mitochondria. Mitochondrion.

[98] Alemany VS, Nomoto R, Saeed MY, Celik A, Regan WL, Matte GS (2023). Mitochondrial transplantation preserves myocardial function and viability in pediatric and neonatal pig hearts donated after circulatory death. J Thorac Cardiovasc Surg..

[99] Guariento A, Blitzer D, Doulamis I, Shin B, Moskowitzova K, Orfany A (2020). Preischemic autologous mitochondrial transplantation by intracoronary injection for myocardial protection. J Thorac Cardiovasc Surg.

[100] Moskowitzova K, Shin B, Liu K, Ramirez-Barbieri G, Guariento A, Blitzer D (2019). Mitochondrial transplantation prolongs cold ischemia time in murine heart transplantation. J Heart Lung Transplant.

[101] Tanaka M, Terry RD, Mokhtari GK, Inagaki K, Koyanagi T, Kofidis T (2004). Suppression of graft coronary artery disease by a brief treatment with a selective epsilonPKC activator and a deltaPKC inhibitor in murine cardiac allografts. Circulation..

[102] Guariento A, Piekarski BL, Doulamis IP, Blitzer D, Ferraro AM, Harrild DM (2021). Autologous mitochondrial transplantation for cardiogenic shock in pediatric patients following ischemia–reperfusion injury. J Thorac Cardiovasc Surg.

[103] Burrowes KS, Clark AR, Tawhai MH (2011). Blood flow redistribution and ventilation-perfusion mismatch during embolic pulmonary arterial occlusion. Pulm Circ.

[104] Van Raemdonck D, Van Slambrouck J, Ceulemans LJ (2022). Donor lung preservation for transplantation-where do we go from here?. J Thorac Dis.

[105] Moskowitzova K, Orfany A, Liu K, Ramirez-Barbieri G, Thedsanamoorthy JK, Yao R (2020). Mitochondrial transplantation enhances murine lung viability and recovery after ischemia–reperfusion injury. Am J Physiol Lung Cell Mol Physiol.

[106] Sommer SP, Sommer S, Sinha B, Wiedemann J, Otto C, Aleksic I (2011). Ischemia–reperfusion injury-induced pulmonary mitochondrial damage. J Heart Lung Transplant.

[107] Cypel M, Yeung JC, Hirayama S, Rubacha M, Fischer S, Anraku M (2008). Technique for prolonged normothermic ex vivo lung perfusion. J Heart Lung Transplant.

[108] Divithotawela C, Cypel M, Martinu T, Singer LG, Binnie M, Chow CW (2019). Long-term outcomes of lung transplant with ex vivo lung perfusion. JAMA Surg.

[109] Ulger O, Kubat GB, Cicek Z, Celik E, Atalay O, Suvay S (2021). The effects of mitochondrial transplantation in acetaminophen-induced liver toxicity in rats. Life Sci.

[110] Ko SF, Chen YL, Sung PH, Chiang JY, Chu YC, Huang CC (2020). Hepatic. J Cell Mol Med.

[111] Lin HC, Liu SY, Lai HS, Lai IR (2013). Isolated mitochondria infusion mitigates ischemia–reperfusion injury of the liver in rats. Shock.

[112] Shi X, Bai H, Zhao M, Li X, Sun X, Jiang H (2018). Treatment of acetaminophen-induced liver injury with exogenous mitochondria in mice. Transl Res.

[113] Kochanek PM, Berger RP, Bayir H, Wagner AK, Jenkins LW, Clark RS (2008). Biomarkers of primary and evolving damage in traumatic and ischemic brain injury: diagnosis, prognosis, probing mechanisms, and therapeutic decision making. Curr Opin Crit Care.

[114] Bambrick L, Kristian T, Fiskum G (2004). Astrocyte mitochondrial mechanisms of ischemic brain injury and neuroprotection. Neurochem Res.

[115] Zhang B, Gao Y, Li Q, Sun D, Dong X, Li X (2020). Effects of brain-derived mitochondria on the function of neuron and vascular endothelial cell after traumatic brain injury. World Neurosurg.

[116] Russo E, Napoli E, Borlongan CV (2018). Healthy mitochondria for stroke cells. Brain Circ.

[117] Zhao J, Qu D, Xi Z, Huan Y, Zhang K, Yu C (2021). Mitochondria transplantation protects traumatic brain injury via promoting neuronal survival and astrocytic BDNF. Transl Res.

[118] Zhang Z, Ma Z, Yan C, Pu K, Wu M, Bai J (2019). Muscle-derived autologous mitochondrial transplantation: a novel strategy for treating cerebral ischemic injury. Behav Brain Res.

[119] Pourmohammadi-Bejarpasi Z, Roushandeh AM, Saberi A, Rostami MK, Toosi SMR, Jahanian-Najafabadi A (2020). Mesenchymal stem cells-derived mitochondria transplantation mitigates I/R-induced injury, abolishes I/R-induced apoptosis, and restores motor function in acute ischemia stroke rat model. Brain Res Bull.

[120] Xie Q, Zeng J, Zheng Y, Li T, Ren J, Chen K (2021). Mitochondrial transplantation attenuates cerebral ischemia–reperfusion injury: possible involvement of mitochondrial component separation. Oxid Med Cell Longev.

[121] Norat P, Soldozy S, Sokolowski JD, Gorick CM, Kumar JS, Chae Y (2020). Mitochondrial dysfunction in neurological disorders: exploring mitochondrial transplantation. NPJ Regen Med.

[122] Doulamis IP, Guariento A, Duignan T, Kido T, Orfany A, Saeed MY (2020). Mitochondrial transplantation by intra-arterial injection for acute kidney injury. Am J Physiol Renal Physiol.

[123] Rossi A, Asthana A, Riganti C, Sedrakyan S, Byers LN, Robertson J (2023). Mitochondria transplantation mitigates damage in an in vitro model of renal tubular injury and in an ex vivo model of DCD renal transplantation. Ann Surg.

[124] Jabbari H, Roushandeh AM, Rostami MK, Razavi-Toosi MT, Shokrgozar MA, Jahanian-Najafabadi A (2020). Mitochondrial transplantation ameliorates ischemia/reperfusion-induced kidney injury in rat. Biochim Biophys Acta Mol Basis Dis.

[125] Arjmand A, Faizi M, Rezaei M, Pourahmad J (2023). The effect of donor rat gender in mitochondrial transplantation therapy of cisplatin-induced toxicity on rat renal proximal tubular cells. Iran J Pharm Res.

[126] Camp KM, Krotoski D, Parisi MA, Gwinn KA, Cohen BH, Cox CS (2016). Nutritional interventions in primary mitochondrial disorders: Developing an evidence base. Mol Genet Metab.

[127] Food and Drug Administration. Critical path innovation meeting regarding drug development for mitochondrial diseases. 2015.

[128] White E, Yeske PE, Gray K, Strittmatter K, Hernandez B, Mann K, et al. Voice of the Patient Report “Mitochondrial Disease: Adults with Myopathy, Children with Neurologic Symptoms”. United Mitochondrial Disease Federation; 2019.

[129] Food and Drug Administration. Developing therapies for primary mitochondrial diseases: bridging the gaps. 2019.

[130] Kaza AK, Wamala I, Friehs I, Kuebler JD, Rathod RH, Berra I (2017). Myocardial rescue with autologous mitochondrial transplantation in a porcine model of ischemia/reperfusion. J Thorac Cardiovasc Surg.

[131] Masuzawa A, Black KM, Pacak CA, Ericsson M, Barnett RJ, Drumm C (2013). Transplantation of autologously derived mitochondria protects the heart from ischemia–reperfusion injury. Am J Physiol Heart Circ Physiol.

[132] Cowan DB, Yao R, Akurathi V, Snay ER, Thedsanamoorthy JK, Zurakowski D (2016). Intracoronary delivery of mitochondria to the ischemic heart for cardioprotection. PLoS ONE.

